# Supplementary material for: Field-level clothianidin exposure affects bumblebees but generally not their pathogens
Source: Nat Commun. 2018 Dec 21;9:5446. doi: 10.1038/s41467-018-07914-3 (PMC6303475; doi:10.1038/s41467-018-07914-3)
Supplement: Supplementary file 1 — Supplementary Information [file 41467_2018_7914_MOESM1_ESM.pdf]

Supplementary Information for

Field-level clothianidin exposure affects bumblebees but generally not their pathogens

Wintermantel *et al.*

Correspondence to: dywintermantel@gmail.com

This PDF file includes:

Supplementary Figure 1

Supplementary Tables 1-4

Supplementary References

Supplementary Figures

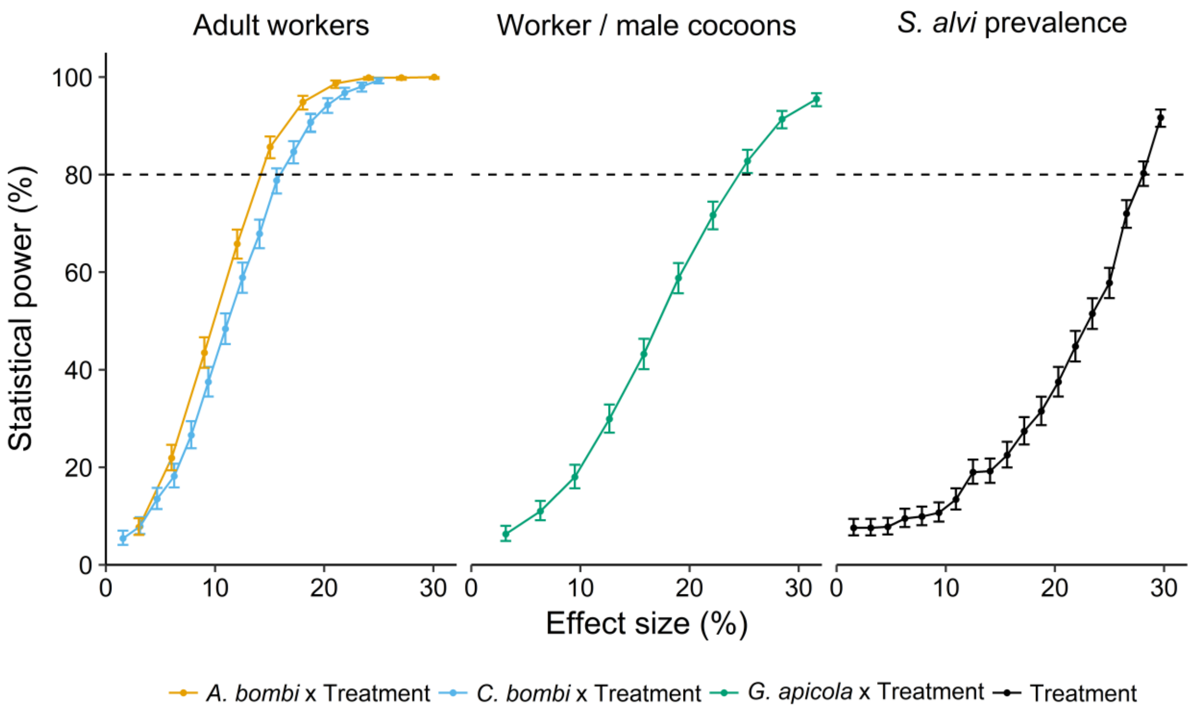

Supplementary Figure 1 | Statistical power. Power in relation to effect size for treatment effects or interactive effects between treatment and microorganism abundance where 0.05<*P*<0.1. For interactive effects on the numbers of adult workers and worker/male cocoons, effect size represents the effect of a $\log_{10}$ unit increase in microorganism abundance expressed as a percentage of the estimated value of a control colony with average microorganism $\log_{10}$ abundance. For *S. alvi* prevalence effect size represents an increase in the number of infected colonies illustrated as a percentage of all colonies. The dashed line indicates a power of 80%.

Supplementary Tables

Supplementary Table 1 | Microorganism prevalence. The prevalences of detected microbiota in adult worker bees were related to clothianidin seed treatment using two different kinds of tests: tests of equal proportions for straight comparisons of the proportions of infected colonies between treatments and analyses of variance (ANOVA) based on the number of infected colonies per field to control for non-independence of colonies placed by the same field.

**Response**	**Predictor**	**Test**	**Estimate** [c]	χ^2_1	***P*** [d]
Acute bee paralysis virus	Treatment	Equal proportions [a]	-9.4%	0.87	0.352
Slow bee paralysis virus	Treatment	Equal proportions [a]	6.3%	1.84	0.668
Sacbrood virus	Treatment	LRT on GLMM [b]	12.2%	1.74	0.189
Apicystis bombi	Treatment	LRT on GLMM [b]	-14.7%	0.71	0.400
Crithidia bombi	Treatment	LRT on GLMM [b]	-20.8%	4.33	**0.037**
Nosema bombi	Treatment	Equal proportions [a]	-9.4%	0.87	0.352
Gilliamella apicola	Treatment	Equal proportions [a]	0%	0	1
Snodgrassella alvi	Treatment	LRT on GLMM [b]	23.8%	3.63	0.057

[a] Two-sided test of equal proportions using the prop.test function in R
[b] Likelihood ratio test on a generalized linear mixed effects model with field identity as random effect.
[c] Effect sizes are expressed as absolute differences between treatments in the percentage of infected colonies.
[d] *P*-values < 0.05 are highlighted in bold

Supplementary Table 2 | Microorganism abundance. Log_{10} abundance of prevalent microorganisms in worker bees of infected colonies (i.e. excluding colonies in which the target organism was not detected) in relation to clothianidin seed treatment [a].

Response	Predictor	Estimate ($\log_{10}$ units)	Estimate (%) [a]	χ^2_1	P [b]	N Fields	N Colonies
A. bombi	Treatment	1.09	24.5	2.39	0.122	15	40
C. bombi	Treatment	- 0.68	- 11.3	2.70	0.100	16	53
G. apicola	Treatment	0.28	5.39	1.39	0.239	16	58
S. alvi	Treatment	0.05	1.10	0.13	0.720	16	50

[a] Effect sizes in % were calculated in reference to the control group
[b] *P*-values were calculated by likelihood ratio tests with 1 degree of freedom on linear mixed effects models containing field identity and field pair identity as random factors.

Supplementary Table 3 | Primers. Forward (F) and reverse (R) primers used in RNA/DNA assays with a literature reference to previous usage.

Target	Primers	Sequence '5-'3
Acute bee paralysis virus[1]	ABPV-F6548 (F)<br>KIABPV-B6707 (R)	TCATACCTGCCGATCAAG<br>CTGAATAATACTGTGCGTATC
Black queen cell virus[1]	BQCV-qF7893 (F)<br>BQCV-qB8150 (R)	AGTGGCGGAGATGTATGC<br>GGAGGTGAAGTGGCTATATC
Chronic bee paralysis virus[1]	CBPV1-qF1818 (F)<br>CBPV1-qB2077 (R)	CAACCTGCCTCAACACAG<br>AATCTGGCAAGGTTGACTGG
Deformed wing virus[1]	DWV-F8668 (F)<br>DWV-B8757 (R)	TTCATTAAAGCCACCTGGAACATC<br>TTTCCTCATTAACTGTGTCG
Lake Sinai Virus type-1[1]	qLSV1-F2569 (F)<br>qLSV1-R2743 (R)	AGAGGTTGCACGGCAGCATG<br>GGGACGCAGCACGATGCTCA
Lake Sinai virus type-2[1]	qLSV2-F1722 (F)<br>qLSV2-R1947 (R)	CGTGCTGAGGCCACGGTTGT<br>GCGGTGTCGATCTCGCGGAC
RNA250[2]	RNA250 (F)<br>RNA250 (R)	TGGTGCCTGGGCGGTAAAG<br>TGCGGGGACTCACTGGCTG
Bt-RPL23[3]	Bt-RPL23 (F)<br>Bt-RPL23 (R)	GGGAAAACCTGAACTTAGGAAAA<br>ACCCTTTCATTTCTCCCTTGTTA
Slow bee paralysis virus[4]	SBPV-F3177 (F)<br>SBPV-B3363 (R)	GCGCTTTAGTTCAATTGCC<br>ATTATAGGACGTGAAAATATAC
Sacbrood virus[1]	SBV-qF3164 (F)<br>SBV-qB3461 (R)	GCTCTAACCTCGCATCAAC<br>TTGGAACTACGCATTCTCTG
Apis mellifera filamentous virus[5]	AmFV-BroN (F)<br>AmFV-BroN (R)	TTATTAACACCGCAGGCTTC<br>CATGGTGGCCAAGTCTTGCT
Gregarine spp.[6, b]	Gregarine (F)<br>Gregarine (R)	CCAGCATGGAATAACATGTAAGG<br>GACAGCTTCCAATCTCTAGTCG
Crithidia bombi [a,b]	Cbombi-GADH-F2 (F)<br>Cbombi-GADH-R2 (R)	CAAGAGCTCGCCGGGT<br>GGACGCGTTCGACACC
Nosema apis [7]	Napis-qF3 (F)<br>NosUniv-qR3 (R)	TAGTATATTTGAATATTTGTTTACAATGG<br>CGCTATGATCGCTTGCC
Nosema ceranae [a, b]	Nceranae-qF3 (F)<br>NosUniv-qR3 (R)	GTATGTTTGAATAATTATTTATTTTATTG<br>CGCTATGATCGCTTGCC
Nosema bombi [a, b]	Nbombi-qF3 (F)<br>NosUniv-qR3 (R)	TAGTATGTTTGAATATTTATTTATTACGA<br>CGCTATGATCGCTTGCC
Gilliamella apicola [a,b]	Gilliam 16S (F)<br>Gilliam 16S (R)	GTAACATGAGTGCTTGCACT<br>CGCATGGCCCGAAGG
Snodgrassella alvi [a,b]	Snodgras 16S (F)<br>Snodgras 16S (R)	ACGGAGAGCTTGCTCTC<br>AAATAACGCGAGGTCTTTCGA

[a] The primers were first used in this study.
[b] Identity of target organism confirmed by bidirectional Sanger sequencing of selected PCR products. In all cases, the PCR product sequences matched 100% with the intended target.

Supplementary Table 4 | Data analysis excluding the Biscaya sprayed field. Tests that resulted in a change of the level of significance (based on α = 0.05) if the field that was sprayed with Biscaya (containing thiacloprid) was removed from the data analysis.

**Response**	**Predictor**	**Sample**	**Estimate**	χ^2_1	***P*** [a]
Crithidia bombi prevalence	Treatment	All colonies	-20.8%	4.33	**0.037**
Crithidia bombi prevalence	Treatment	Excluding Biscaya sprayed field	-20.1%	3.63	0.057
Worker/male cocoons	*Apicystis bombi*	All colonies	-8.946	3.70	0.054
Worker/male cocoons	*Apicystis bombi*	Excluding Biscaya sprayed field	-14.174	5.08	**0.024**

[a] *P*-values are based on likelihood ratio test on a generalized linear mixed effects model with field identity as random effect.

Supplementary References

1. Locke, B., Forsgren, E., Fries, I. & de Miranda, J. R. Acaricide treatment affects viral dynamics in *Varroa destructor*-infested honey bee colonies via both host physiology and mite control. *Appl. Environ. Microbiol.* **78,** 227–235 (2012).
2. Mondet, F., de Miranda, J. R., Kretzschmar, A., Le Conte, Y. & Mercer, A. R. On the Front Line: Quantitative Virus Dynamics in Honeybee (Apis mellifera L.) Colonies along a New Expansion Front of the Parasite Varroa destructor. *PLoS Pathog.* **10,** e1004323 (2014).
3. Niu, J., Cappelle, K., de Miranda, J. R., Smagghe, G. & Meeus, I. Analysis of reference gene stability after Israeli acute paralysis virus infection in bumblebees *Bombus terrestris*. *J. Invertebr. Pathol.* **115,** 76–79 (2014).
4. de Miranda, J. R. *et al.* Genetic characterization of slow bee paralysis virus of the honeybee (*Apis mellifera* L.). *J. Gen. Virol.* **91,** 2524–2530 (2010).
5. Hartmann, U., Forsgren, E., Charrière, J. D., Neumann, P. & Gauthier, L. Dynamics of *Apis mellifera* filamentous virus (AmFV) infections in honey bees and relationships with other parasites. *Viruses* **7,** 2654–2667 (2015).
6. Meeus, I., De Graaf, D. C., Jans, K. & Smagghe, G. Multiplex PCR detection of slowly-evolving trypanosomatids and neogregarines in bumblebees using broad-range primers. *J. Appl. Microbiol.* **109,** 107–115 (2010).
7. Forsgren, E. & Fries, I. Comparative virulence of *Nosema ceranae* and *Nosema apis* in individual European honey bees. *Vet. Parasitol.* **170,** 212–217 (2010).
