# Supplementary material for: Field-level clothianidin exposure affects bumblebees but generally not their pathogens
Source: Nat Commun. 2018 Dec 21;9:5446. doi: 10.1038/s41467-018-07914-3 (PMC6303475; doi:10.1038/s41467-018-07914-3)
Supplement: Supplementary file 3 — Description of Additional Supplementary Files [file 41467_2018_7914_MOESM3_ESM.docx]

**Description of Additional Supplementary Files**

**File Name**: Supplementary Data 1

**Description**: The co-variation of microorganism abundance in adult workers.

**File Name:** Supplementary Data 2.

**Description**: The interactive effect between clothianidin exposure and microorganism

abundance on bumblebee performance.
