# Supplementary material for: Field-level clothianidin exposure affects bumblebees but generally not their pathogens
Source: Nat Commun. 2018 Dec 21;9:5446. doi: 10.1038/s41467-018-07914-3 (PMC6303475; doi:10.1038/s41467-018-07914-3)
Supplement: Supplementary file 6 — Reporting summary [file 41467_2018_7914_MOESM6_ESM.pdf]

nature research

Corresponding author(s): Dimitry Wintermantel

Reporting Summary

Nature Research wishes to improve the reproducibility of the work that we publish. This form provides structure for consistency and transparency in reporting. For further information on Nature Research policies, see Authors & Referees and the Editorial Policy Checklist.

Statistical parameters

When statistical analyses are reported, confirm that the following items are present in the relevant location (e.g. figure legend, table legend, main text, or Methods section).

n/a	Confirmed	
☐	☒	The exact sample size (*n*) for each experimental group/condition, given as a discrete number and unit of measurement
☐	☒	An indication of whether measurements were taken from distinct samples or whether the same sample was measured repeatedly
☐	☒	The statistical test(s) used AND whether they are one- or two-sided<br>*Only common tests should be described solely by name; describe more complex techniques in the Methods section.*
☐	☒	A description of all covariates tested
☐	☒	A description of any assumptions or corrections, such as tests of normality and adjustment for multiple comparisons
☐	☒	A full description of the statistics including central tendency (e.g. means) or other basic estimates (e.g. regression coefficient) AND variation (e.g. standard deviation) or associated estimates of uncertainty (e.g. confidence intervals)
☐	☒	For null hypothesis testing, the test statistic (e.g. *F*, *t*, *r*) with confidence intervals, effect sizes, degrees of freedom and *P* value noted<br>*Give P values as exact values whenever suitable.*
☒	☐	For Bayesian analysis, information on the choice of priors and Markov chain Monte Carlo settings
☐	☒	For hierarchical and complex designs, identification of the appropriate level for tests and full reporting of outcomes
☒	☐	Estimates of effect sizes (e.g. Cohen's *d*, Pearson's *r*), indicating how they were calculated
☐	☒	Clearly defined error bars<br>*State explicitly what error bars represent (e.g. SD, SE, CI)*

Our web collection on statistics for biologists may be useful.

Software and code

Policy information about availability of computer code

Data collection	No software was used to collect data.
Data analysis	All data were analysed in R version 3.3.4.

For manuscripts utilizing custom algorithms or software that are central to the research but not yet described in published literature, software must be made available to editors/reviewers upon request. We strongly encourage code deposition in a community repository (e.g. GitHub). See the Nature Research guidelines for submitting code & software for further information.

Data

Policy information about availability of data

All manuscripts must include a data availability statement. This statement should provide the following information, where applicable:

- Accession codes, unique identifiers, or web links for publicly available datasets
- A list of figures that have associated raw data
- A description of any restrictions on data availability

The data supporting the findings of this study are available within the paper, its supplementary information files and/or Rundlöf et al.17. The datasets generated during and/or analysed during the current study are available from the corresponding author on reasonable request.

Field-specific reporting

Please select the best fit for your research. If you are not sure, read the appropriate sections before making your selection.

☐ Life sciences ☐ Behavioural & social sciences ☒ Ecological, evolutionary & environmental sciences

For a reference copy of the document with all sections, see nature.com/authors/policies/ReportingSummary-flat.pdf

Ecological, evolutionary & environmental sciences study design

All studies must disclose on these points even when the disclosure is negative.

Study description	The study followed A randomized controlled design with paired fields of which one field per pair was randomly assigned to be sown with Elado-treated oilseed rape seeds, while the other field was sown with insecticide-free oilseed rape seeds. Two sets of three bumblebee colonies were placed in protective houses at each field. The outer 2 colonies in each set were used in this study, giving a total of 4 colonies per field.
Research sample	We investigated commercially reared bumblebee (Bombus terrestris L.) colonies and their individual bees. The colonies may be considered representative of wild bees with intermediate level of sociality.
Sampling strategy	We sampled 64 colonies out of 96 that were placed. From each colony we sampled 10 adult female workers and 10 pupae of each caste (where available) for pathogen analyses, which represents roughly 5-10% of the total colony size.
Data collection	Data on colony development measures were collected by a postdoctoral student and an assistant. The microbial data were collected by authors of this manuscript (Dimitry Wintermantel & Emilia Semberg) as well as the measures of individual bee size and developmental stage (Dimitry Wintermantel).
Timing and spatial scale	The colonies were placed at 16 fields in Southern Sweden between June 14-28 2013 and all colonies of one field pair were freeze-killed simultaneously between July 7th and August 5th 2013, at first sighting of new queens in any of those 12 colonies. The colonies were afterwards weighed, dissected and bees of different castes were counted. The laboratory analysis was mostly conducted in the summer of 2015.
Data exclusions	We excluded ~0.7% of adult bees from the analysis because they could not be categorized into one of the castes. Among the adult worker bumblebees used for the microbial analysis, 7 (i.e. 1.1%) were excluded from the analysis of body mass and intertegular distance as they lacked a body part.
Reproducibility	We did not repeat this experiment.
Randomization	Fields were paired according to geographical location and land use in their surroundings. Treatments were randomly assigned to fields within a pair. Colonies were randomly allocated to the fields.
Blinding	Pseudonyms were used to mask treatment groups for people collecting data.

Did the study involve field work? ☒ Yes ☐ No

Field work, collection and transport

Field conditions	The colonies were placed in readily accessible locations on the edge of commercial oilseed rape farms in the southern part of Sweden, all with permission FROM the landowners. The experimental period WAS May-AUGUST 2013.
Location	The experiment was conducted in southern Sweden. The approximate locations of the oilseed rape fields are given in the manuscript.
Access and import/export	Only bumblebees from experimentally placed, commercially reared colonies were sampled. These were imported to Sweden from the producers in the Netherlands in compliance with intra-EU trade and movement of animals.
Disturbance	No major disturbance was caused by the placement of bumblebee colonies in agricultural landscapes.

Reporting for specific materials, systems and methods

Materials & experimental systems

n/a	Involved in the study
☒	☐ Unique biological materials
☒	☐ Antibodies
☒	☐ Eukaryotic cell lines
☒	☐ Palaeontology
☐	☒ Animals and other organisms
☒	☐ Human research participants

Methods

n/a	Involved in the study
☒	☐ ChIP-seq
☒	☐ Flow cytometry
☒	☐ MRI-based neuroimaging

Animals and other organisms

Policy information about studies involving animals; ARRIVE guidelines recommended for reporting animal research

Laboratory animals	None
Wild animals	None
Field-collected samples	Male and female bumblebees (Bombus terrestris L.) at different life stages (pupae and adults) from commercially produced colonies placed near commercial oilseed rape cultivations.
